# Virtual stimulation of the interictal EEG network localizes the EZ as a measure of cortical excitability

**DOI:** 10.3389/fnetp.2024.1425625

**Published:** 2024-08-20

**Authors:** Sophia R. Zhai, Sridevi V. Sarma, Kristin Gunnarsdottir, Nathan E. Crone, Adam G. Rouse, Jennifer J. Cheng, Michael J. Kinsman, Patrick Landazuri, Utku Uysal, Carol M. Ulloa, Nathaniel Cameron, Sara Inati, Kareem A. Zaghloul, Varina L. Boerwinkle, Sarah Wyckoff, Niravkumar Barot, Jorge A. González-Martínez, Joon Y. Kang, Rachel June Smith

**Affiliations:** ^1^ Department of Biomedical Engineering, Johns Hopkins University, Baltimore, MD, United States; ^2^ Institute for Computational Medicine, Johns Hopkins University, Baltimore, MD, United States; ^3^ Department of Neurology, Johns Hopkins University, Baltimore, MD, United States; ^4^ Department of Neurosurgery, University of Kansas Medical Center, Kansas City, KS, United States; ^5^ Department of Neurology, University of Kansas Medical Center, Kansas City, KS, United States; ^6^ Surgical Neurology Branch, National Institute of Neurological Disorders and Stroke, National Institutes of Health, Bethesda, MD, United States; ^7^ Barrow Neurological Institute, Phoenix Children’s Hospital, Phoenix, AZ, United States; ^8^ Department of Neurology, University of Pittsburgh, Pittsburgh, PA, United States; ^9^ Department of Neurosurgery, University of Pittsburgh, Pittsburgh, PA, United States; ^10^ Department of Electrical and Computer Engineering, University of Alabama at Birmingham, Birmingham, AL, United States

**Keywords:** epileptogenic zone, dynamic network model, intracranial EEG, virtual stimulation, cortical excitability, single-pulse electrical stimulation, cortico-cortical evoked potentials

## Abstract

**Introduction:** For patients with drug-resistant epilepsy, successful localization and surgical treatment of the epileptogenic zone (EZ) can bring seizure freedom. However, surgical success rates vary widely because there are currently no clinically validated biomarkers of the EZ. Highly epileptogenic regions often display increased levels of cortical excitability, which can be probed using single-pulse electrical stimulation (SPES), where brief pulses of electrical current are delivered to brain tissue. It has been shown that high-amplitude responses to SPES can localize EZ regions, indicating a decreased threshold of excitability. However, performing extensive SPES in the epilepsy monitoring unit (EMU) is time-consuming. Thus, we built patient-specific *in silico* dynamical network models from interictal intracranial EEG (iEEG) to test whether virtual stimulation could reveal information about the underlying network to identify highly excitable brain regions similar to physical stimulation of the brain.

**Methods:** We performed virtual stimulation in 69 patients that were evaluated at five centers and assessed for clinical outcome 1 year post surgery. We further investigated differences in observed SPES iEEG responses of 14 patients stratified by surgical outcome.

**Results:** Clinically-labeled EZ cortical regions exhibited higher excitability from virtual stimulation than non-EZ regions with most significant differences in successful patients and little difference in failure patients. These trends were also observed in responses to extensive SPES performed in the EMU. Finally, when excitability was used to predict whether a channel is in the EZ or not, the classifier achieved an accuracy of 91%.

**Discussion:** This study demonstrates how excitability determined via virtual stimulation can capture valuable information about the EZ from interictal intracranial EEG.

## Introduction

Medically refractory epilepsy (MRE), also known as drug-resistant epilepsy or pharmacoresistant epilepsy, refers to a condition where seizures persist despite treatment with appropriate antiepileptic medications (Kwan and Brodie, 2010). This condition affects 30 percent of individuals with epilepsy, making it a challenging and often debilitating neurological disorder (Kwan and Sander, 2004). For individuals with focal epilepsy and identifiable Epileptogenic zones (EZs), surgical resection may offer the potential for seizure freedom. Comprehensive presurgical evaluation, including neuroimaging studies, extensive EEG monitoring, and neuropsychological assessment, is essential for identifying suitable candidates for surgery and precisely localizing the EZ (Rosenow and Lüders, 2001).

Localization of the EZ is challenging and time-dependent (Enatsu and Mikuni, 2016; Burneo et al., 2006). Neurologists may never capture a seizure during scalp and even intracranial EEG monitoring (Pondal-Sordo et al., 2007). Surgical success rates vary from 30% to 70% because no clinically validated biomarker of the EZ currently exists (Mohan et al., 2018; Jobst and Cascino, 2015). It has been shown, however, that highly epileptogenic regions often display increased levels of cortical excitability, which can be probed using single-pulse electrical stimulation (SPES) by delivering brief pulses of electrical current to the brain tissue (Hays et al., 2022; Smith et al., 2022; Frauscher et al., 2023). Specifically, the resulting cortico-cortical evoked potentials (CCEPs) display larger amplitudes in epileptogenic regions (Iwasaki et al., 2010; Enatsu et al., 2012; Matsumoto et al., 2017; Hays et al., 2022). These observations suggest that performing extensive SPES across the brain with intracranial EEG electrodes could aid in more accurately localizing the EZ (Hays et al., 2022). However, performing SPES is time consuming, and this creates a barrier to the implementation of SPES in the clinic.

We hypothesize that EEG recording site pairs exhibiting strong causal relationships are more prone to activation during seizures (Smith et al., 2022; Gunnarsdottir et al., 2022; Li et al., 2019), and we further believe this connection can be measured from baseline interictal activity. Subsequently, we hypothesize that a metric of cortical excitability derived from *virtual stimulation* of patient-specific *in silico* models will provide information on the EZ location, aiding in localizing areas to remove for seizure control. To test this hypothesis, a dynamical network model (DNM) is constructed from sequential 500 ms windows of interictal intracranial EEG (iEEG), similar in duration to the trials that occur in SPES (Hays et al., 2023; Hays et al., 2021). To mimic SPES, a perturbation of a 1 ms pulse is applied to the DNM to produce a simulated time series. Then, the norm is calculated across each channel’s time series to quantify its “excitability.” Excitability is compared between the clinically annotated EZ and non-EZ brain regions. We found that the EZ channels responses showed higher excitability than non-EZ regions regardless of which brain region is virtually stimulated in patients with successful surgical outcomes. Additionally, we found that this trend diminishes with decreasing success in surgical outcome. A similar pattern is found in true CCEPs that were recorded for a subset of patients.

## Methodology

### Data collection: intracranial EEG recordings

Intracranial interictal EEG data [combination of stereoelectroencephalography (sEEG) and electrocorticography (ECoG)] was collected from 69 epilepsy patients that were evaluated at five epilepsy centers (Johns Hopkins University, the Cleveland Clinic, the University of Kansas Medical Center, the National Institutes of Health, and the University of Pittsburgh Medical Center) and assessed for clinical outcome 1 year after surgery. The data were recorded with either a Nihon Kohden (Nihon Kohden America, LLC, Irvine, CA, United States) or a Natus (Natus Medical, Inc., Middleton, WI, United States) recording system. Data were sampled between 500 and 2 kHz.

### Data collection: patient population

The patient population is summarized in Tables 1 and 2.

**TABLE 1:** Dataset demographic.

	CC	KUMC	JHU	NIH	UPMC	Total
Number of patients	31	9	15	9	5	69
Gender, male/female	15/16	4/5	5/10	7/2	3/2	34/35
Age, years	30.23 ± 11.98	39.67 ± 16.87	33.93 ± 12.52	33.11 ± 9.27	36.6 ± 11.99	33.10 ± 12.57
Surgical outcome, S/F	14/17	4/5	8/7	4/5	3/2	33/36
Duration of interictal recordings (sec)	238.63 ± 218.98	601.88 ± 2.23	849 ± 248.70	300 ± 0	308.96 ± 6.43	421.41 ± 311.52

**TABLE 2:** Engel scores.

	Engel 1	Engel 2	Engel 3	Engel 4
All patients	32	21	9	7
Patients with CCEP Data	5	3	6	0

### Data collection: clinical annotations of the EZ

At each epilepsy center, the clinical team independently formulated an EZ hypothesis based on the data collected during presurgical evaluation for each patient, which included both non-invasive and invasive imaging techniques.

The centers utilize qualitative assessments of the iEEG to define regions of seizure onset, which remains the gold standard. Clinicians at the participating institutions employ a combination of non-invasive and invasive techniques to localize the EZ. At all centers, a comprehensive evaluation is performed and consensus is reached using imaging (MRI, PET, SPECT, and MEG at most centers), semiology, scalp EEG, and intracranial EEG, with common iEEG seizure onset patterns such as low voltage fast activity and rhythmic sharp activity being critical (Perucca et al., 2014). The spatial and temporal evolution include criteria of evolving low frequency high amplitude periodic/rhythmic spikes, rhythmic sharply contoured activity less than beta range, and 2–4 Hz spike and wave activity which shows evolution. These patterns should occur before any clinical changes and are foundational for localizing the epileptogenic zone. This encompasses sEEG channels that show the earliest electrophysiological changes, typically characterized by low voltage fast activity, at the onset of a seizure event (i.e., channels corresponding to the seizure onset zone), as well as channels involved in the early spread of the seizure. There were no restrictions on seizure onset patterns. All of these methods are reviewed in interdisciplinary meetings to ensure agreement on the localization of the EZ.

The clinically annotated EZ (CA-EZ) refers to the specific anatomical area(s) designated for treatment (such as resection, ablation, or stimulation). The criteria for CA-EZ are anatomoelectroclinical in nature. EZ channels were clinically annotated in patients with both successful and failed surgical outcomes.

### Clinical classification of surgical outcomes

Each epilepsy center’s epileptologists categorized surgical outcomes according to the Engel Surgical Outcome Scale. An Engel score (from 1 to 4) is assigned to each patient depending on the clinical response to surgical treatment. Engel score of 1 indicates that seizure freedom is achieved while Engel score of 4 indicates no worthwhile improvement is observed (J, 1993). Successful outcomes were defined as being free of disabling seizures or rare occurrence (less than 3 seizure days per year) at 12 or more months post-operation (Engel class 1 and 2), while failure outcomes indicated the persistence of disabling seizures (Engel classes 3 and 4) (J, 1993). Out of 94 patients, 70 achieved successful outcomes, while 24 continued to experience disabling seizures after treatment.

### Data preprocessing

Interictal data segments were clipped with starting times defined at random at least 24 h prior to seizure. The clips were 5–10 min long, and included mostly wake, but some sleep periods. As described in our earlier work (Gunnarsdottir et al., 2022), the iEEG data underwent bandpass filtering between 0.5 and 300 Hz using a fourth order Butterworth filter, and notch filtering at 60 Hz and its harmonics with a stopband of 2 Hz. The applied bandpass filter included up to 300 Hz, so gamma/high gamma activity was included in the signals that we used to construct our models. We uniformly applied all data preprocessing. We re-referenced the data to the common average to eliminate common noise from the signals and visually removed excessively artifactual channels. Electrode locations were determined by combining information from co-registered post-implantation CT and brain MRI scans (e.g., using BioImage Suite). The clinical team at each center visually confirmed the electrode localizations for accuracy. Subsequently, sEEG channels not recording from grey matter or deemed “bad” by clinicians (e.g., located in white matter, broken, excessively noisy, or artifactual) were excluded from each patient’s dataset, resulting in an average of 102 ± 34 (mean ± SD) channels used per patient in the analysis.

The sEEG recordings were segmented into non-overlapping 500 ms windows for modeling and feature extraction (details below). All data processing and analysis were conducted using MATLAB R2020b.

### Virtual stimulation of a DNM

**FIGURE 1:**
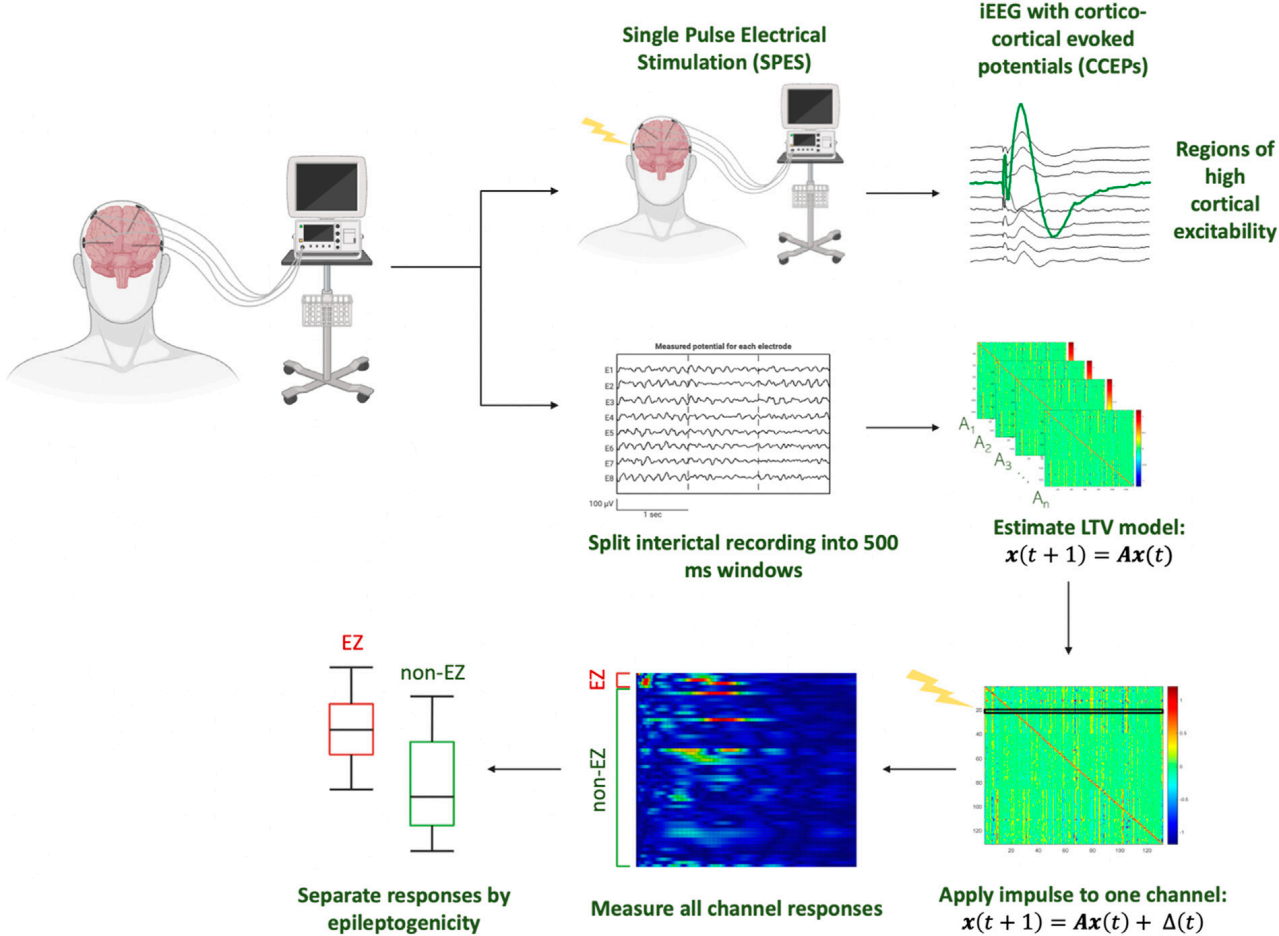
Analysis pipeline from iEEG data, modeling responses of simulated perturbations to the DNM constructed from interictal iEEG data of individual patients.

To virtually mimic SPES from interictal data, a patient-specific DNM is constructed by approximating dynamics as linear behavior in discrete, short time-intervals (Smith et al., 2022; Gunnarsdottir et al., 2022; Li et al., 2019; Smith et al., 2020; Kamali et al., 2020) (Figure 1). The DNM is a generative model capturing the dynamic influences of each iEEG channel on the rest of the network as well as how each channel is influenced by the other channels. Previous work demonstrates how DNMs accurately reconstruct the iEEG data (Li et al., 2017; Li et al., 2021). These models can then be virtually perturbed to simulate how SPES perturbs the brain with electrical stimulation.

To do this, the data were first divided into non-overlapping 500 ms windows, and a linear time-invariant model of the form:

$$x\left(t+1\right)=Ax\left(t\right)$$ (1)

was constructed for each window (Gunnarsdottir et al., 2022). For a given patient, let N be the number of iEEG channels. In this model, A ∈ ℝ^NxN^ is the state transition matrix and x(t) ∈ ℝ^N^ represents the iEEG channels (Eq. 1). The A matrix describes channel interaction from the iEEG and these interactions’ evolution over time. Specifically, the element A_ij_ describes how the present activity of channel j influences the future activity (next sample) of channel i. Thus, the i^th^ row generally contains information on the cumulative effect of the entire network on the i^th^ channel. The j^th^ column contains dynamics of how the j^th^ channel influences other channels.

After the A matrices are estimated, an exogenous perturbation was then added for each channel:

$$x_{stim}\left(t+1\right)=Ax_{stim}\left(t\right)+\Delta \left(t\right)$$ (2)

where Δ is the discrete time signal pulse of width 1 ms applied to the model in the first millisecond. Next, the network response time series, $x_{stim}\left(t\right)$ , was generated (Eq. 2) for 50 time steps with a zero initial condition (Figure 1). The perturbation is applied at the beginning for each A matrix, paralleling the clinical SPES trials which are often collected with 1 Hz periodic stimulation. A pulse width of 1 ms and simulation duration of 50 ms was chosen through an optimization procedure (Supplementary Figure S1).

### Quantifying channel excitability

Since $x_{stim}\left(t\right)$ is a multivariate time series of the predicted response, we further derived a metric for quantifying channel excitability. For each **A** matrix, we do the following:

1. Apply a pulse perturbation to one channel and generate the response $x_{stim}\left(t\right)$ .
2. Compute the 2-norm (also known as the Euclidean distance and defined as the square root of the inner product of a vector with itself) of the time series to quantify excitability for each channel.
3. Normalize each channels’ 2-norm to a range from 0 to 1.
4. Repeat steps 1–3 for all channels to produce an excitability matrix (**E**) where the rows represent the channel in which the simulated response is quantified, and the columns represent the stimulated channel.

Subsequently, each **E** matrix element is identified as one of four categories depending on the stimulated and responding channel: stimulated in CA-EZ and response in CA-EZ (EZ:EZ), stimulated in CA-EZ and response in non-CA-EZ (EZ:non-EZ), stimulated in non-CA-EZ and response in CA-EZ (non-EZ:EZ), stimulated in non-CA-EZ and response in non-CA-EZ (non-EZ:non-EZ). For each category of stimulation and response type outlined above, we calculate the average of all elements in the category. Finally, excitability of the EZ is computed as the average of the values from EZ:EZ and non-EZ:EZ. Excitability of the non-EZ is calculated by taking the average of the values from EZ:non-EZ and non-EZ:non-EZ.

**FIGURE 2:**
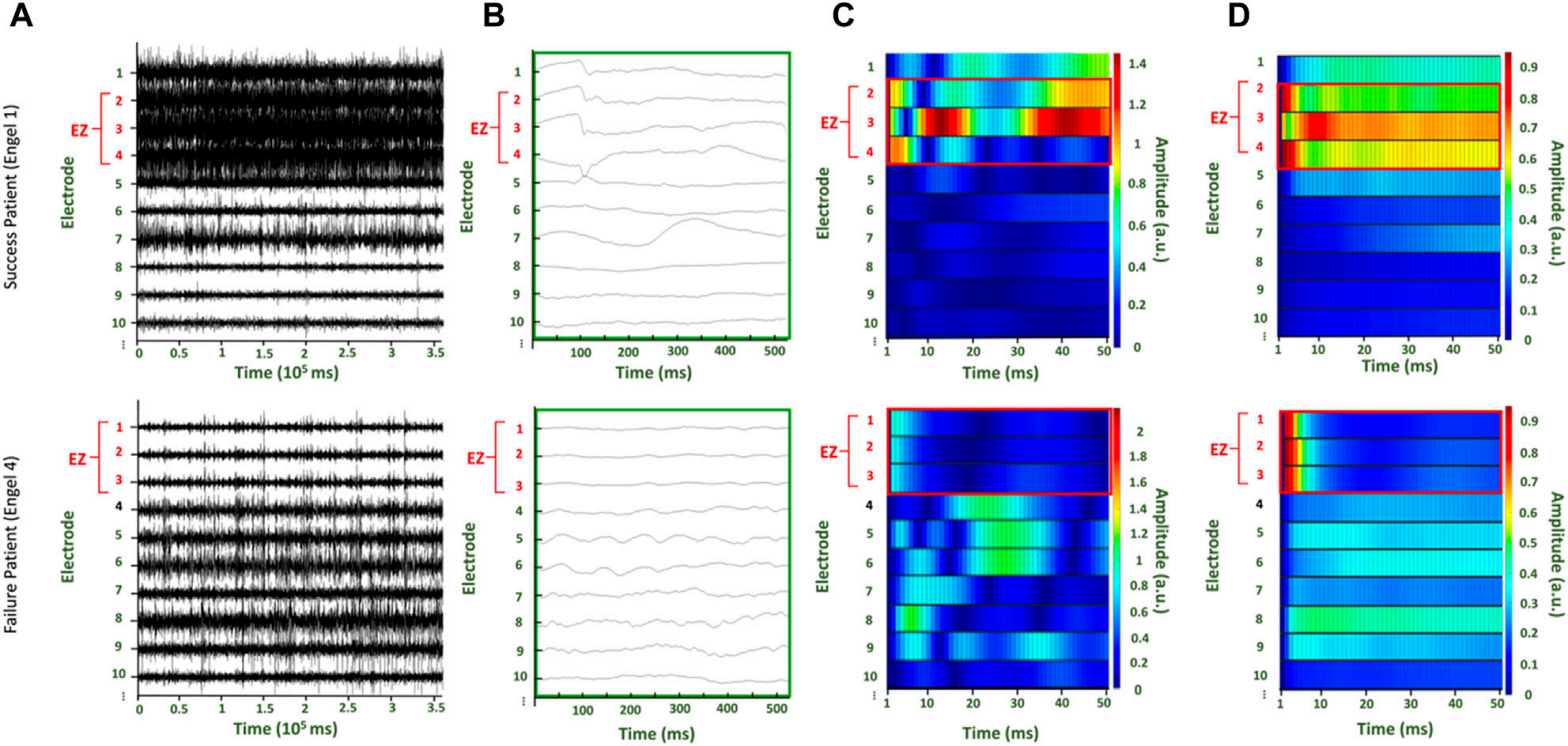
Two patients with surgical outcomes of Engel score 1 (top row) and Engel score 4 (bottom row), respectively. **(A)** Raw interictal iEEG data collected for the patients **(B)** Time series data from the first 500 ms of the raw interictal iEEG from which the first A matrix is estimated **(C)** The simulated time series from the first trial, where the first A matrix is perturbed with unit pulse in the first channel **(D)** The average of the time series from all trials after each time point is normalized to the maximum value.

Thus, each patient had two values that define the excitability of EZ and non-EZ regions, regardless of the stimulation location. Patients were categorized as success or failure patients, defined as Engel Scores of 1 and 2 vs. 3 and 4, respectively. The EZ and non-EZ excitabilities were first Figures 2C, D compared in Engel 1 patients, where it is most likely that the EZ was localized correctly. Then, the difference was compared between success and failure patients as well as between individual Engel scores. Significance was determined by the Wilcoxon rank-sum test.

### SPES response analysis

In a subset of patients undergoing intracranial EEG monitoring at Johns Hopkins Hospital, SPES was performed as part of a research protocol. Bipolar stimulation was administered in adjacent pairs of contacts at a 0.5 Hz stimulation frequency. The stimulation current ranged from 3 to 7 mA with a 300 us pulse width. Stimulation was applied to all channels that were localized in brain tissue. During post-processing, stimulation artifact was removed by assessing the value of the signal 2 ms before stimulation and 6 ms after stimulation and creating a linear vector in between the two points (Smith et al., 2022). As the N1 amplitude was calculated between 10 and 60 ms after the stimulus, we found that there was often negligible residual artifact in this time frame.

The excitability distributions across EZ and non-EZ channels were compared to excitabilities computed from true CCEPs recorded during SPES as the objective is to assess whether virtual stimulation provides similar clinical information from interictal data as SPES. The average response waveform for each channel over a 2 s window where the stimulus starts 0.5 s is calculated. Then, the RMS of the CCEPs were calculated and normalized over the maximum RMS. Similar to the virtual stimulation paradigm, responses were separated into EZ vs. non-EZ responses and stratified between success or failure patients. Since the SPES was performed in electrode pairs, if either electrode that was stimulated was located in the CA-EZ, then it was considered to be stimulated in the EZ.

### Logistic regression prediction of EZ channels in Engel 1 patients

To assess whether there is congruence between our most excitable channels as identified through virtual stimulation and the clinically annotated EZ, we wanted to focus on Engel 1 patients where the ground truth of the EZ is likely close to the clinically annotated EZ (this ensures we use the most reliable labels for the regression). We tested whether cortical excitabilities derived from virtual stimulation could predict which nodes belonged to the CA-EZ. For Engel 1 patients, the virtual stimulation excitabilities were randomly split into training and validation sets at a 75:25 ratio for 10-fold validation. A logistic regression model was fit to the training set and used to predict EZ and non-EZ channels in the test set. Accuracy and AUC of the predictions were calculated. The average and variance of the accuracy and AUC were determined, and ROC curves were generated.

## Results

### Regions exhibiting high cortical excitability during virtual stimulation correspond to CA-EZ in patients with successful surgeries

For each patient, the raw EEG data (Figure 2A) is segmented in 500ms windows (Figure 2B) to build a DNM. A response is produced for each perterbation of each A matrix. The perturbation-elicited response can be visualized in the format of a heat map (Figures 2C, D). Each row is the response of an electrode to the perturbation. Simulations of the response were generated for 50 ms (Supplementary Figure S1). In the representative patients, the CA-EZ correlates to the channels with visibly greater excitability values in the surgical success (top row) than the surgical failure (bottom row) (Figure 2). For both patients, the CA-EZ correlated with channels that have greater values for the average across all trials of the normalized time series (Figure 2D).

### Higher cortical excitability of EZ compared to non-EZ in Engel 1 patients

**FIGURE 3:**
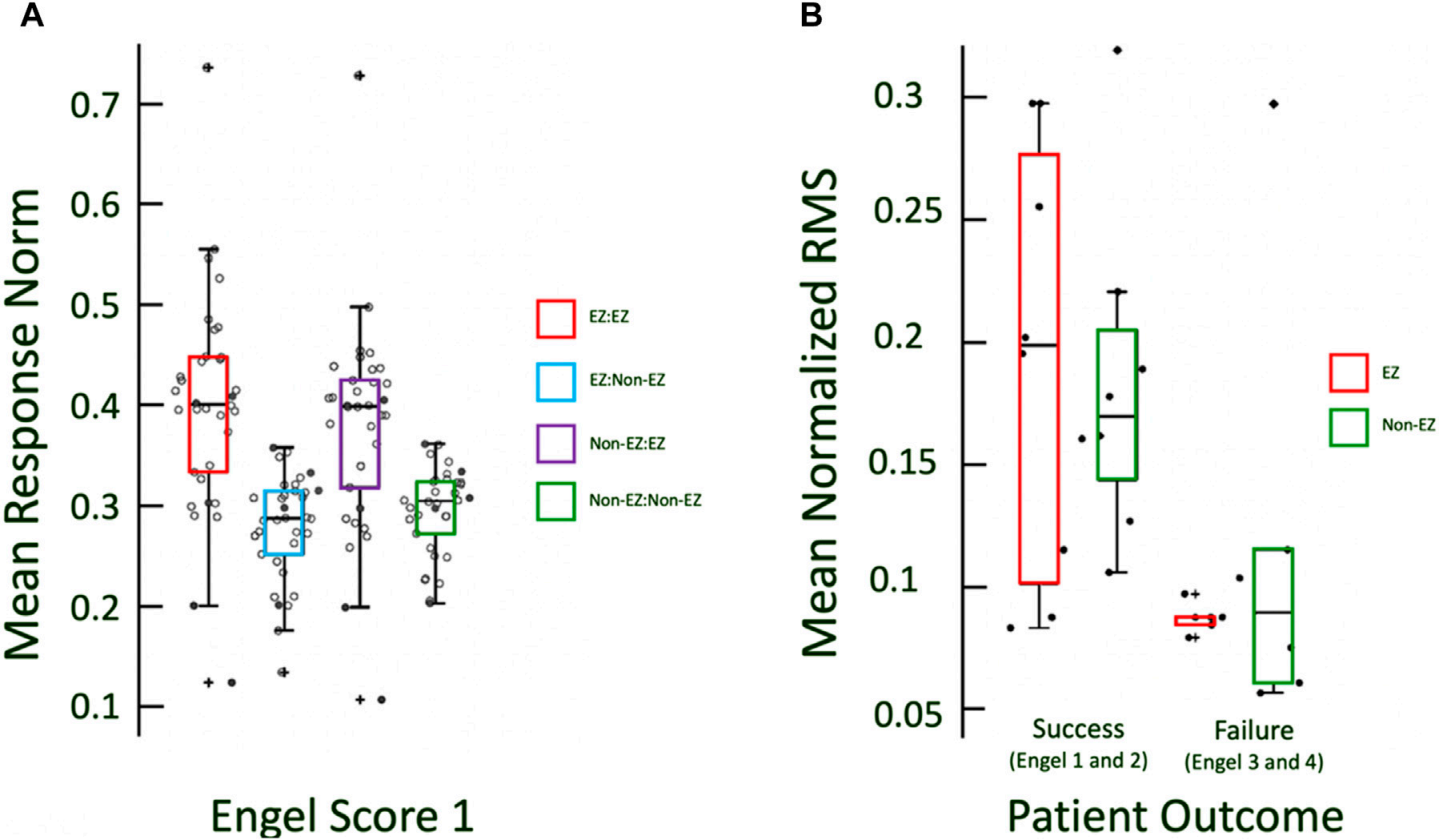
**(A)** Boxplots of excitabilities of EZ and non-EZ regions for Engel 1 patients. Excitability in CA-EZ is higher than non-CA-EZ regardless of the origin of stimulation. Filled in dots indicate the Engel 1 patients for which CCEP data was recorded. **(B)** RMS of responses is marginally greater in CCEPs for EZ regions in success outcome patients and not in non-EZ.

We found that regions that were labeled as the CA-EZ by the respective clinical team displayed high levels of cortical excitability when compared to regions outside the CA-EZ (*p* < 0.05, Wilcoxon rank-sum test) in patients that were Engel 1 (Figure 3A). The higher excitability in CA-EZ regions occurs for both stimulation from CA-EZ regions and stimulation from non-CA-EZ regions. The subset of patients labeled Engel 1 that were also evaluated with SPES are indicated with filled dots (Figure 3).

### Greater difference in CA-EZ excitability in successful patient outcomes

**FIGURE 4:**
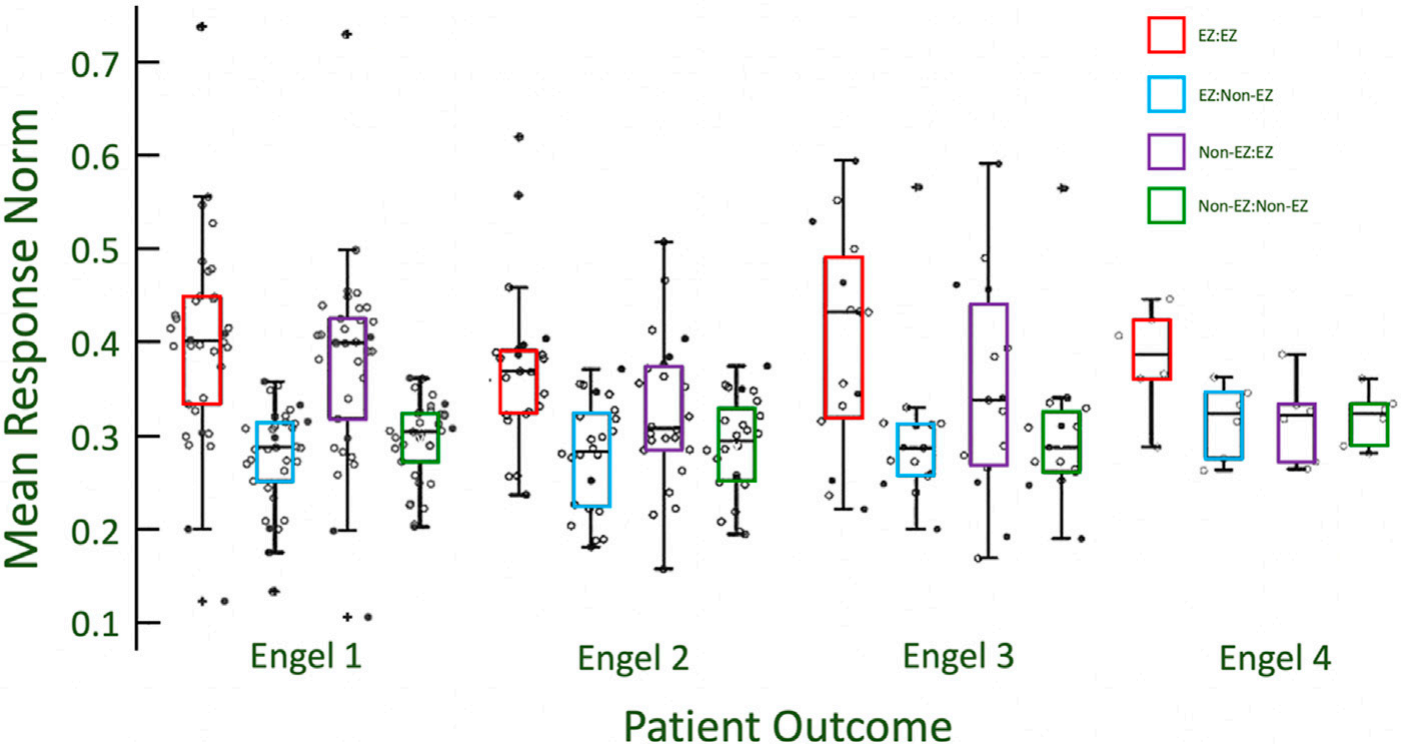
The stratification of excitability between EZ and non-EZ regions is greater for patients with more successful outcomes. Engel 1 has the unique results that show that CA-EZ excitability is higher regardless of what channels/regions are being stimulated. This is seen by comparing the red and purple box plots to the blue and green box plots within each class. With poorer outcome classes, the excitability of the CA-EZ excitability remains high when stimulating from CA-EZ regions, but is comparable to non-CA-EZ excitability when stimulating from non-CA-EZ.

We found that stratification of excitability between CA-EZ and non-CA-EZ increased with better treatment outcomes (Figure 4). The more successful the outcome was, the larger the difference in excitability values of the CA-EZ and non-CA-EZ channels. Engel 1 patients demonstrated the most significant difference in excitability of EZ versus non-EZ channels (Wilcoxon rank sum, *p* < 0.05) (Figure 4). Thus, evoked responses have increased amplitude when the response node is in an epileptogenic region. It is noted that for all four Engel scores, there is elevated excitability in the CA-EZ when the CA-EZ is stimulated. However, the excitability of CA-EZ regions from stimulation of non-CA-EZ channels is not significantly different from non-CA-EZ excitability for patients with failed outcomes (Wilcoxon rank sum test, *p* > 0.05).

It is noted that Engel class 3 EZ responses have a higher distribution than for Engel class 1 when virtually stimulated from EZ channels. However, the EZ responses of Engel class 3 when stimulated from non-EZ channels are not higher than that of Engel class 1 and 2 (Figure 4). This may imply that a defining characteristic of EZ channels is the high response after virtual stimulation from non-EZ channels in addition to other EZ nodes.

### CCEPs demonstrate similar trend to virtual stimulation excitabilities

A similar trend of cortical excitability, as measured by the RMS of the CCEP evoked potential, occurred in the CCEP responses, with successful patients exhibiting a slightly greater mean response in CA-EZ channels compared to non-CA-EZ channels (Figure 3B). The patients with Engel scores 3 and 4, in contrast, did not exhibit significant differences in responses (Wilcoxon rank sum, *p* > 0.05). It is noted that the mean normalized RMS values of failure patients, whether CA-EZ or non-CA-EZ responses, are significantly lower than those of success patients (Figure 3).

The factors of patient gender, patient age, numbers of electrodes, and previous surgery status were assessed by comparing corresponding excitabilities (EZ:EZ, EZ:non-EZ, non-EZ:EZ, non-EZ:non-EZ) between the various categories, and no difference in excitabilities were found associated with these factors (Supplementary Figure S2).

### Logistic regression differentiates EZ and non-EZ channels from virtual stimulation excitabilities in Engel 1 patients

**FIGURE 5:**
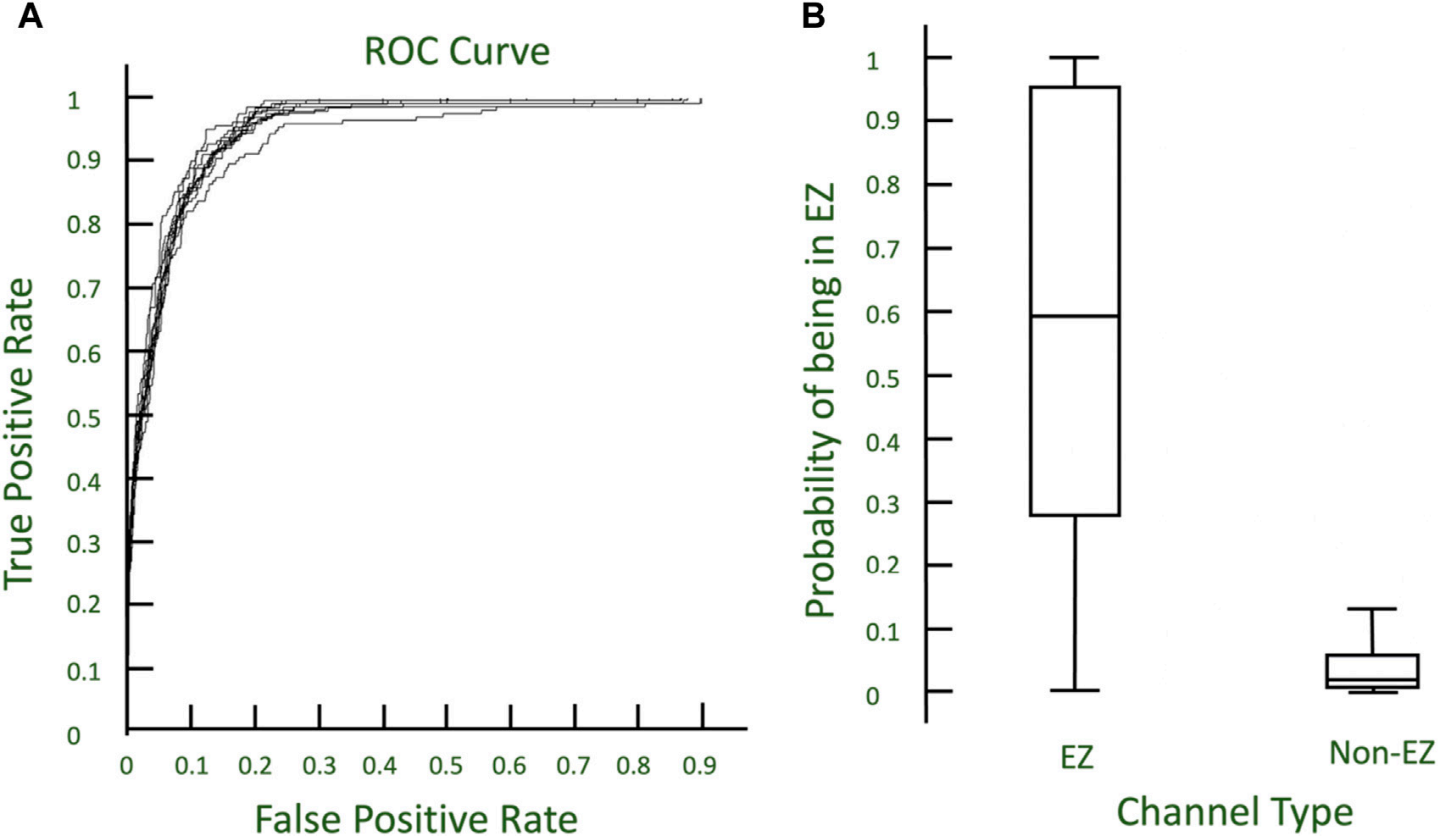
**(A)** ROC curves describing the 10-fold cross validation of logistic regression models to predict EZ nodes. **(B)** Boxplot of probabilities from models predicting EZ and non-EZ channels.

Excitability from virtual stimulation in each channel in CA-EZ and non-CA-EZ regions were calculated. Across 10 iterations, training a logistic regression model of randomly selected 75% of all the channels across the Engel 1 patients yielded a mean accuracy of 91.4243% with variance 0.2155% when applied on the remaining 25% of channels (Figure 5). The ROC curve demonstrates that the model accurately identifies channels in the EZ utilizing two excitability values, one from stimulating EZ regions and one from stimulating non-EZ regions. The mean AUC was 0.95047 with variance 5.8292e-05 (Figure 5). Thus, the excitability of a specific channel from stimulating the EZ vs. non-EZ can predict whether the channel is in the EZ or not.

## Discussion

### Clinical relevance

The increased response of labeled EZ regions in patients with successful outcomes makes excitability a promising metric for EZ localization. The differences in excitability of the EZ and non-EZ labeled regions indicates that the interictal iEEG may provide revealing information of epileptic cortical network dynamics to help effectively pinpoint locations that should be removed to control seizures. While EZ channels demonstrate elevated excitability from virtual stimulation of EZ channels across all Engel scores, the virtual stimulation of non-EZ channels results in greater excitability for patients with good surgical outcomes. On the contrary, poor outcome cases do not have greater excitability of EZ channels from virtual stimulation of non-EZ channels. Thus, a defining characteristic of EZ channels may be the greater sensitivity to virtual stimulation from non-EZ channels.

Virtual perturbation may be revealing information on the underlying network dynamics that complement the clinical information captured in CCEPs from SPES. Additionally, the stratification by outcome demonstrates that this metric aligns with current methods of successful localization since the labels by clinicians from continuous monitoring in patients with successful outcomes are significantly more excitable than the channels that are labeled as non-EZ. It is important to note there are only 14 patients with CCEP data.

We did not expect CCEPs responses to necessarily resemble virtual responses because in CCEPs many more brain regions outside implantation or coverage may influence the responses (Li et al., 2019; Li et al., 2017). Thus, we hypothesized that patients with fewer channels and/or coverage of perhaps the most involved regions would show more correspondence between virtual and CCEPs responses (Supplementary Figure S3).

Furthermore, determining excitability is supportive of previous hypotheses proposed of source-sink connectivity for fingerprinting the EZ (Gunnarsdottir et al., 2022). While the mechanism for onset of seizure is unclear, a virtual stimulation may result in propagation to areas that are highly excitable and reactive. These highly excitable regions are likely the EZ.

Our results aligned well with clinicians’ labeling of EZ regions in patients with successful outcomes and less so in unsuccessful patients. This may be indicative that EZ regions were unidentified or inaccurate in the localization for patients of Engel score 3 and 4 (Figure 4). However, we note there were some high values of excitabilities in EZ channels in the failed outcome patients (Figure 4). This result suggests the clinicians may be localizing some or all of the EZ spread accurately, even in patients with poor surgical outcomes. Failure may be due to unmeasured larger network activation or not being able to treat the identified regions if they are in eloquent cortex.

Calculating excitability from clips of interictal data is fast and efficient compared to continuous monitoring which is the current method of EZ localization (Enatsu and Mikuni, 2016; Burneo et al., 2006). Our results show that based on the interictal data utilized by the model, we can to some extent replicate the identification of EZ contacts done by clinicians through extensive monitoring which includes both ictal and interictal iEEG, seizure semiology, and preoperative imaging. This tool can be used to inform neurologists how to pinpoint the EZ. In the future, this may be utilized to reduce reliance on capturing seizures in the hospital and ultimately improve surgical outcomes.

The utility of this approach lies in its ability to enhance EZ localization during intracranial iEEG monitoring. By capturing a short clip of interictal data, a linear time-varying model can be constructed, enabling virtual stimulation to identify the most excitable channels. These identified channels can then be compared to clinical annotations. If there is congruence between the most excitable channels and the clinically annotated EZ, then virtual stimulation can bolster confidence in the localization of the EZ. Conversely, if the most excitable channels do not align with the clinically annotated EZ, clinicians can re-evaluate the iEEG data at these excitable channels, which were not initially considered regions of interest. This new information could potentially improve surgical outcomes. Virtual stimulation may be an independent predictive biomarker in the future, but this would need to be confirmed in a comprehensive surgical outcome study.

### Limitations and future direction

The information about the EZ that can be gleaned from virtual stimulation as well as SPES relies on the electrode coverage in each patient. The implantation map varies for each patient and electrodes are placed in different cortical areas. It is possible that there is a greater possibility of connectivity and heightened response if two electrodes are closer geographically. Applying a perturbation to an area of electrodes close in geographical proximity can be utilized to understand if perturbations to subsets of electrodes result in changed excitabilities. Future work entails incorporating spatial information between electrodes to inform excitability. Finally, understanding how clinical factors, such as variability in workflow, anti-epileptic medication doses, and resources across clinics, affect both CCEP responses and overall outcomes could improve how electrical stimulation is used in the epilepsy clinic.

Since our method does not consider spatial information, but rather captures dynamic functional connections, we have eliminated the potential confound of localized stimulation artifact. CCEPs within a close range of the stimulation location frequently have to be disregarded due to stimulation artifact leakage (often within 1 cm of stimulation location). The virtual stimulation method does not need to remove close local connections because no stimulation artifact exists. The benefit of having no stimulation artifact is a facet of this project that we would like to further quantify in future work. Furthermore, the functional connections are likely to show similar inverse relationships as stimulation-evoked effective connections (Crocker et al., 2021; Paulk et al., 2022), which could be examined in future work.

In this study, we concentrated on interictal periods when patients were not taking anti-seizure medications (ASMs). Should we utilize interictal iEEG data from periods during which clinicians adjusted ASMs in the initial days of the epilepsy monitoring unit (EMU) stay, we could incorporate the effects of these medications into the model as an “exogenous” input. This consideration is reserved for future research.

Since there are different synchrony dynamics in wakefulness compared to sleep, it will also be of interest to determine if findings vary between wake and sleep samples. The sleep/wake labels for our data were not readily available as some of the clinicians have left their original institution and this is a secondary analysis of data from another study (Gunnarsdottir et al., 2022). Since sleep/wake criteria were not specified as criteria across all centers, our data likely includes clips from both wakefulness and sleep, which is a limitation of our study. More work is necessary to compare virtual stimulation of wake versus sleep dynamics.

In future surgical outcome studies, we will investigate if the findings are consistent between subpopulations of epilepsy types. A higher diagnostic resolution with more detailed clinical information will allow us to determine whether the methods have increased accuracy for mesial temporal compared to neocortical or extratemporal sites.

## Conclusion

Epileptic cortical network dynamics can be investigated with less invasive and time-consuming methods by utilizing virtual stimulation of dynamic network models constructed from interictal iEEG. The excitability of a simulated perturbation to the epileptogenic network was greatest for patients with successful surgical outcomes and diminished for patients with failed outcomes. Complementary clinical information was found for virtual stimulation evoked responses as true corticocortical evoked potentials from SPES.

Thus, an *in silico* model of SPES built from interictal iEEG data provides a complementary view to traditional SPES and provides insight into local cortical excitability. Our results closely aligned with the clinicians’ labeling of EZ regions, with large differences in excitability between the EZ and non-EZ regions in surgical success patients.

## Data Availability

The raw data supporting the conclusions of this article will be made available by the authors, without undue reservation.
