# Supplementary material for: Virtual stimulation of the interictal EEG network localizes the EZ as a measure of cortical excitability
Source: Front Netw Physiol. 2024 Aug 20;4:1425625. doi: 10.3389/fnetp.2024.1425625 (PMC11368849; doi:10.3389/fnetp.2024.1425625)
Supplement: Supplementary file 1 [file Image1.pdf]

Supplementary Material

Optimization procedure

To determine the virtual stimulation parameters, we performed a parameter sweep to find which parameters separated EZ and non-EZ excitability most significantly in Engel 1 patients. The pulse width, $\Delta(t)$, and duration of simulation were determined through the following optimization procedure:

1. The pulse width was varied from 1 to 150 time steps
2. The analysis window was varied from 50 to 250 time steps
3. For each $\Delta(t)$ and window size pairing, we measured the cortical excitability in EZ and non-EZ regions in all Engel 1 patients
4. The z-score of the median EZ distribution compared to the non-EZ distribution was calculated to quantify which virtual input and response duration separated the two groups most significantly.

In patients with Engel score 1 outcomes, median EZ and non-EZ cortical excitability was the most significantly different when a unit pulse was applied, and excitability was measured over 50ms (50 time steps). The determined parameters of 1 ms pulse width and 50 ms simulation duration were utilized throughout the analysis. Interestingly, the 50 ms time coincides with the duration of N1 response of CCEP.

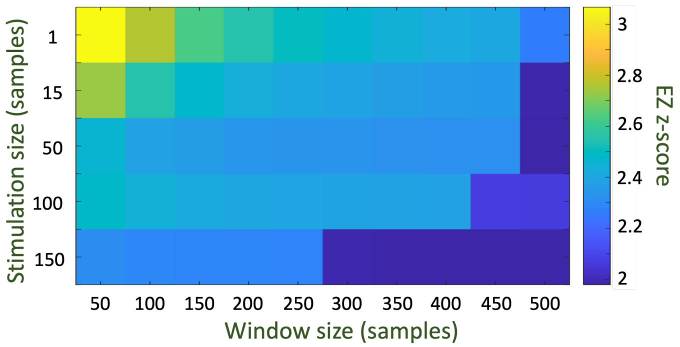

Supplementary Figure 1. Z-score heatmap of pulse duration and window size optimization when median EZ value was compared to non-EZ disribution.

Excitability separation robust to patient gender, age, number of electrodes, and previous surgery

We found that cortical excitability as measured via virtual stimulation is robust across varying numbers of electrodes, patient gender, patient age, or if previous surgery had been performed for the patient. Distributions of excitabilities were not significantly different between category types ($p >> 0.05$).

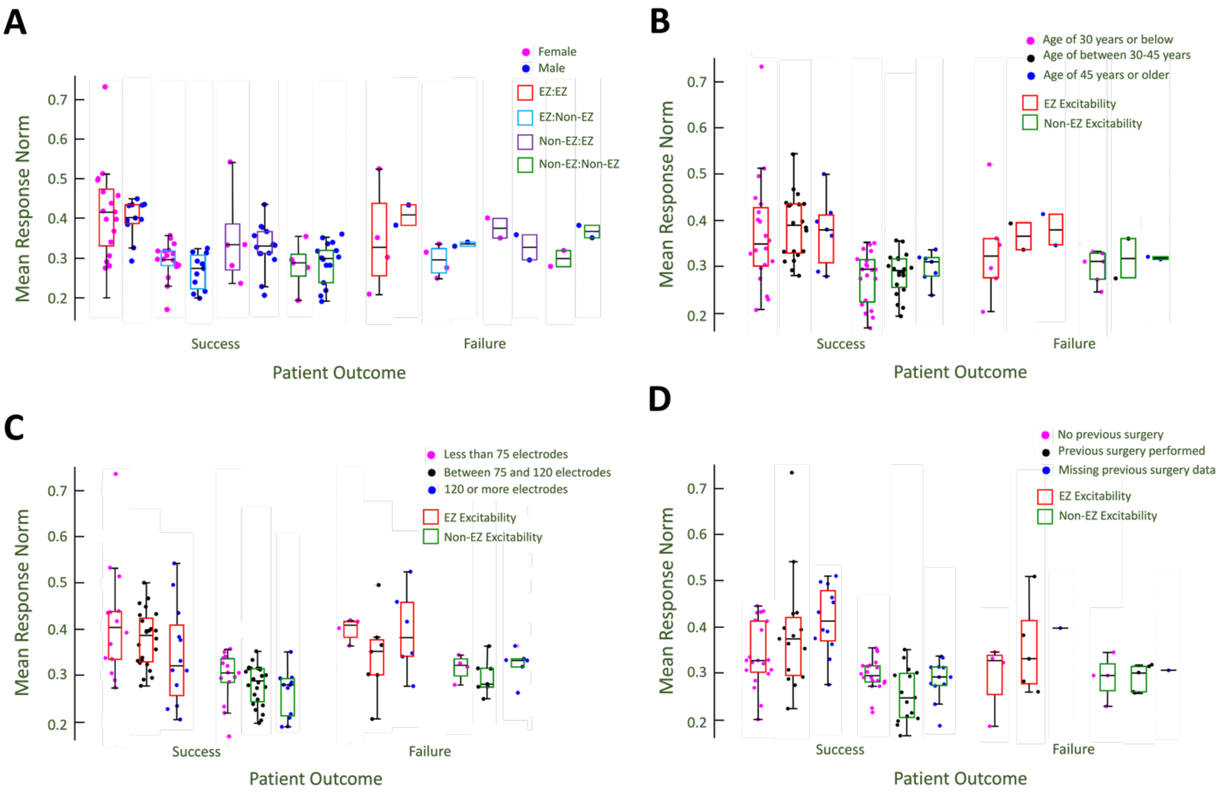

Supplementary Figure 2: Excitability metric is robust to (A) patient gender, (B) patient age, (C) numbers of electrodes, and (D) previous surgery status. EZ excitability remains higher than non-EZ excitability for all groups. The elevated excitability of EZ compared to non-EZ is notable in all patient subgroups and the excitability values between corresponding groups (number of electrodes, record of previous surgery, etc.) were not significantly different.

CCEP N1 RMS values compared to virtual responses

To evaluate the correlation between virtual stimulation and CCEPs, we identified the top 50% most responsive channels with the highest CCEP N1 RMS values. Then, we measured the excitability from virtual stimulation in the same subset of channels. From the 14 patients, we excluded two with abnormally large CCEPs RMS values. We have plotted the CCEP/virtual responses in two separate categories, one with more than 115 channels recorded for the patient (n=4), and one with less than 115 channels recorded (n=8).

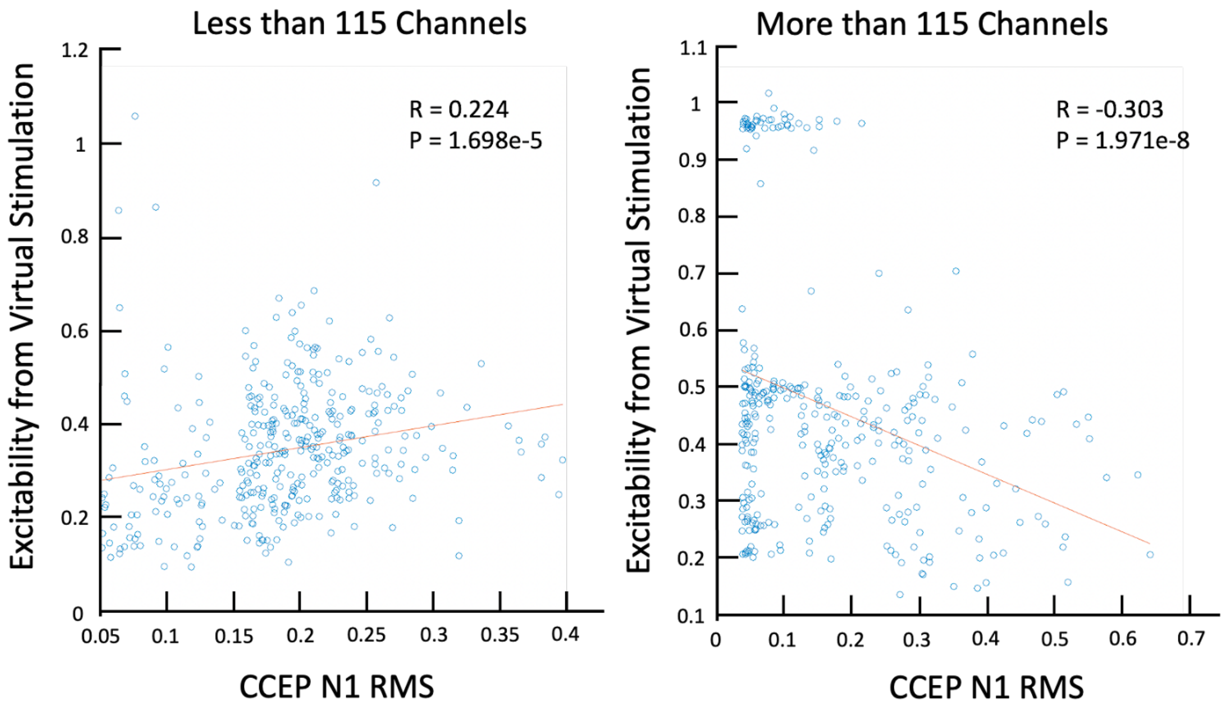

Supplementary Figure 3: There is a significant positive correlation between CCEP N1 RMS and excitability from virtual stimulation for the 50% highest responding channels in the patients with less than 115 channels recorded. There is a negative correlation for patients with more than 115 channels recorded.
